# Prevalence and Predictors of Internet Addiction among College Students in Sousse, Tunisia

**Published:** 2017-01-02

**Authors:** Menel Mellouli, Nawel Zammit, Manel Limam, Mariem Elghardallou, Ali Mtiraoui, Thouraya Ajmi, Chekib Zedini

**Affiliations:** ^1^Department of Family and Community Medicine, Faculty of Medicine of Sousse, Tunisia

**Keywords:** Internet, Behavior-addictive, Students, Tunisia

## Abstract

**Background:** Internet represents a revolution in the world of technology and communication all over the world including Tunisia. However, this technology has also introduced problematic use, especially among students. The current study aimed to determine the prevalence of Internet addiction among college students and its predictors in the region of Sousse, Tunisia.

**Study design:** A cross-sectional study.

**Methods:** The current study was conducted in the colleges of Sousse, Tunisia in 2012-2013. A self-administrated questionnaire was used to collect data from 556 students in 5 randomly selected colleges from the region. Collected data concerned socio-demographic characteristics, substances use and internet addiction using the Young Internet Addiction Test.

**Results:** The response rate was 96%. The mean age of participants was 21.8 ±2.2 yr. Females represented 51.8% of them. Poor control of internet use was found among 280 (54.0%; 95% CI: 49.7%, 58.3%) participants. Low education levels among parents, the young age, lifetime tobacco use and lifetime illicit drugs use were significantly associated with poor control of internet use among students (*P*<0.001). While, the most influential factor on internet use among them was under-graduation with an adjusted odds ratio of 2.4 (95% CI: 1.7, 3.6).

**Conclusions:** Poor control of internet use is highly prevalent among the college students of Sousse especially those under graduate. A national intervention program is required to reduce this problem among youth. A national study among both in-school and out-of-school adolescents and young people would identify at-risk groups and determine the most efficient time to intervene and prevent internet addiction.

## Introduction

Internet represents a revolution in the world of technology and changed the world in term of communication. In 2013, more than the third of the world’s population and almost all college students were using the internet ^1^. However, this "new" technology has also introduced, especially among young people, problematic use such as addiction to online gaming, gambling, chatting and pornographic videos watching^2^. Since the 1990s, internet addiction has been recognized as a mental health problem similar to the other established addictions^3^. Nevertheless, internet addiction was not officially recognized as a disorder by the psychiatric community^4^. In addition, there were various tools and cutoff points to measure the addiction levels and thus, there was a wide range of reports on the prevalence rate of internet addiction among youth^5,6^.

Internet addiction can cause several issues including psychosocial, academic, occupational and financial difficulties besides health problems such as carpal tunnel syndrome, dry eyes, neck muscular problems, headaches and sleeping problems^1^. Internet addiction depends on personal characteristics. While the environmental and the socio-cultural factors seem to have a great influence on internet use^7,8^. In Tunisia, the internet use rose from 17% in 2008 to 52.1% in 2016^9^. In 2014, 45% of Tunisian people over 18 yr had computer in their households, 25% had landline in their houses, 12% had smartphone and 77% were accessing the internet daily^10^. In the education sector, the Tunisian internet agency reported 100% connectivity including universities ^11^. Nevertheless, few studies were led to explore internet addiction among youth in Tunisia.

This study aimed to determine the level of internet addiction and its predictors among college students in Sousse, Tunisia. Better understanding of such emergent mental health problems in Tunisia could place them on the national public agenda and catalyze prevention actions at the national and local levels.

## Methods

### Study design

This cross-sectional study was conducted in the region of Sousse, Tunisia in 2012-2013 among college students in order to determine the prevalence and the predictors of addictive behaviors among them.

### Study population

Sample size calculation was based on an expected prevalence of addictive behaviors of 13% based on a previous study ^12^, a precision of 3% and confidence level of 95% which gave a required sample of at least 483 patients. Considering an attrition rate of 20%, a sample of 580 was estimated. For this, a list of the 17 colleges of the region was composed. Then, the colleges were divided into 5 fields of study: Economy, Arts, Letters, Health, and Technology. From each specialty, one faculty was randomly selected. The 5 colleges obtained were as follows: the Institute of Fiscal Studies, the Institute of Music, the Faculty of Law, the Faculty of Medicine and the Higher Institute of Applied Sciences and Technology. Students enrolled in these 5 colleges, who were present in the premises of the campuses after the completion of a course, a lecture or a break near to the classrooms, the lecture theaters, the refreshment bars or in the gardens during the days of data collection were approached and asked for participation.

### Data collection

Anonymous questionnaire served to collect data. Each participant filled it up separately after giving verbal explanation by a pre-trained investigator. Collected data concerned socio-demographic characteristics, substances use, and internet use. Use of illicit drug, tobacco, and alcohol were defined as never used or ever used (lifetime use) with ever use divided into regular use (at least once per week during at least 1 month) and irregular use (less than once per week) for illicit substances and alcohol use ^13^. Concerning tobacco, participants were asked about its daily use during the last month. The tools used to measure the cannabis, alcohol, and tobacco addictions were respectively:

The Cannabis Abuse Screening Test (CAST), a 6-item scale assessing the frequency of the following events within the past 12 months: on recreational use (2 questions), memory disorders, being encouraged to reduce or stop using cannabis, unsuccessful attempts to quit, problems linked to cannabis consumption. All items were answered on a 5-point scale (0 “never”, 1 “rarely”, 2 “from time to time”, 3 “quite often”, and 4 “very often”). Then the original five-point scale was dichotomized differentiating “never” versus any occurrence of these events in the past year. Using this algorithm, individual test scores ranged from 0 to 6. Low addiction level was considered for people who obtained one, moderate level was for those who obtained scores of 2 and high level was for those who obtained at least 3^14^.

The Fast Alcohol Consumption Evaluation questionnaire (FACE), a 5 questions interview, for each gender the questionnaire contains two distinct cutoffs that separate nondependent heavy drinkers from the ‘‘low-risk group’’ and the ‘‘dependence group’’. Accordingly, scores <5, between 5 and 8 and >8 indicate low risk, heavy drinking, and alcohol abuse respectively for men and scores <4, between 4 and 8 and >8 indicate low risk, heavy drinking and alcohol abuse respectively for women^15^.

Two questions from the Fagerström Tolerance Questionnaire and the Fagerström Test for Nicotine Dependence about the number of cigarettes smoked per day and the time to the first cigarette after waking were used to obtain a six-point scale (The Heaviness of Smoking Index (HSI)). Nicotine dependence was then categorized into a three-category variable: low (0–1), medium (2–4) and high (5–6) ^16^.

The tool used to measure internet use was the Young Internet Addiction Test (IAT) developed by Kimberly Young^17^. It has an excellent internal consistency reliability and face and constructs validity in addition to an adequate concurrent validity^18^. It consists of 20 items questionnaire answered in a 6 options Likert scale: (0: does not apply, 1: rarely, 2: occasionally, 3: frequently, 4: often, 5: always). It explores the degree to which internet use affects: daily routine, social life, productivity, sleeping pattern, and feelings. The minimum score is 0, and the maximum is 100. The higher the score, the greater the level of addiction is. The obtained scores were categorized into four categories: no addiction (0-30), mild dependence (31-49), moderate dependence (50-79) and severe dependence (80-100)^19^. Scores upper than 50, indicate poor control of internet use ^20^.

### Data analyses

Data were analyzed using SPSS Statistics for Windows, ver. 18.0 (SPSS Inc., Chicago, IL, USA). Quantitative variables were presented as means with standard deviations (SD), and qualitative ones as absolute frequencies and percentages. Differences between groups were examined using Student’s *t* -test to compare means and using Chi-square and Fisher’s exact tests to compare proportions. The dependent variable "Internet addiction score ≥ 50" was coded in two categories (yes and no). All associated factors to this variable that were significant at the 20% level were included in a multivariable model. Then, a stepwise backward approach was used to select the independent variables significantly associated with poor control of internet use for the final model. Results of logistic models were expressed as odds ratios (ORs) with confidence level (CI) of 95%. All statistical tests were 2-tailed, and p values <0.05 were considered statistically significant.

In the univariable and multivariable analysis, information about the dependent variable “poor control of internet use” was missing for 38 participants. Accordingly, their correspondent observations were omitted.

### Ethical considerations

This study was undertaken with respect to the rights and integrity of people. Permissions were obtained from the Ethical Committee of the Faculty of Medicine of Sousse and from the regional Higher Education authorities.

Besides, verbally informed consents were obtained from all participants. Participation was voluntary, and the participants did not receive any payment or supervision. Confidentiality and anonymity were ensured by coding data collection sheets.

## Results

The responses of 556 college students were obtained (participation rate of 96%). The mean age of participants was 21.8 (±2.2) yr. Females represented 51.8% of them. The mean IAT score was 51.4 (±14.6). Concerning the control of internet use, 42 of respondents (8.1%; 95% CI: 5.8%, 10.4%) had no features of internet addiction, 196 (37.8%; 95% CI: 33.6%, 42.0%) had mild level of internet addiction, 262 (50.6%; 95% CI: 46.3%, 54.9%) had moderate level of internet addiction and 18 (3.5%; 95% CI: 1.9%, 5.1%) had severe level of internet addiction. Otherwise, 280 (54.0%; 95% CI: 49.7%, 58.3%) of participants had poor control of internet use (scores of IAT upper than 50).

Univariate analysis revealed that participants with IAT upper than 50, were significantly younger (21.5 ±2 yr) than those with IAT lower than 50 (22.1 ±2.3 yr) (*P* =0.001). Besides, a low parental educational level was significantly more common among students with IAT upper than 50 than among those with IAT lower than 50 (Table 1). Concerning the other risk behaviors, unlike alcohol use, lifetime tobacco use and lifetime illicit drug use were significantly associated with the poor control of internet use (Table 1). The association between poor control of internet use and lifetime illicit drug use was stronger (OR=2.33 95% CI: 1.01, 5.36) than its association with lifetime tobacco use (OR=1.59; 95% CI: 1.03, 2.45) (Table 1).

**Table 1:** Control of internet use according to the individual characteristics of the participants from the colleges of Sousse, Tunisia in 2012-2013 school year

**Characteristics**	**Internet addiction score**		**Unadjusted OR (95% CI)**
	**≥50(n=280)**	**<50(n=238)**	
**Age (yr)**	21.5 (2.0)	22.1 (2.3)	0.87 (0.79, 0.94)
**Gender**			
Female	139 (49.6)	136 (57.1)	1.00
Male	141 (50.4)	102 (42.9)	1.35 (0.95, 1.91)
**Field of study**			
Health	55 (19.6)	62 (26.1)	1.00
Law	68 (24.3)	63 (26.5)	1.22 (0.74, 2.00)
Economy	33 (11.8)	22 (9.2)	1.69 (0.88, 3.24)
Technology	114 (40.7)	87 (36.6)	1.47 (0.93, 2.33)
Arts	10 (3.6)	4 (1.7)	2.82 (0.84, 9.49)
**Degree level**			
Graduate degree	96 (34.5)	132 (55.5)	1.00
Undergraduate degree	182 (65.5)	106 (44.5)	2.36 (1.65, 3.36)
**Academic success**			
Yes	250 (89.6)	218 (91.6)	1.00
No	29 (10.4)	20 (8.4)	1.26 (0.69, 2.29)
**Location**			
Rural	220 (79.4)	184 (77.3)	1.00
Urban	57 (20.6)	54 (22.7)	1.13 (0.74, 1.72)
**Single**			
No	68 (24.5)	56 (23.6)	1.00
Yes	210 (75.5)	181 (76.4)	0.95 (0.64, 1.43)
**Socioeconomic level**			
High	25 (9.0)	19 (8.0)	1.00
Medium	240 (86.3)	212 (89.5)	0.86 (0.46, 1.60)
Low	13 (4.7)	6 (2.5)	1.65 (0.53, 5.13)
**Mother is jobless**			
No	78 (30.7)	59 (26.0)	1.00
Yes	176 (69.3)	168 (74.0)	0.79 (0.53, 1.18)
**Father’s educational level**		
Secondary school/ university level	43 (17.6)	57 (26.5)	1.00
Illiterate/ primary school	201 (82.4)	158 (73.5)	1.68 (1.07, 2.64)
**Mother’s educational level**		
Secondary school/ university level	100 (40.2)	117 (53.2)	1.00
Illiterate/ primary level	149 (59.8)	103 (46.8)	1.69 (1.17, 2.44)
**Parental marital status**			
Married	265 (96.7)	228 (97.9)	1.00
Divorced/widow	9 (3.3)	5 (2.1)	1.54 (0.51, 4.69)
**Current residential status**		
Alone or sharing with friends	71 (25.4)	62 (26.2)	1.00
With family	208 (74.6)	175 (73.8)	1.04 (0.69, 1.54)
**Substances use**			
Lifetime tobaccouse			
No	212 (75.7)	198 (83.2)	1.00
Yes	68 (24.3)	40 (16.8)	1.59 (1.03, 2.45)
Tobaccouse during the last month			
No	21 (32.8)	16(41.0)	1.00
Yes	43 (67.2)	23(59.0)	1.03 (0.31, 3.40)
Tobacco dependence			
Low	39 (63.9)	26 (66.7)	1.00
Moderate	20 (32.8)	12 (30.8)	1.11 (0.46, 2.65)
High	2 (3.3)	1 (2.6)	1.33 (0.11, 15.47)
Alcohol lifetime use			
No	234 (83.6)	209 (87.8)	1.00
Yes	46 (16.4)	29 (12.2)	1.12 (0.44, 2.89)
Alcohol regular use			
No	26 (57.8)	18 (62.1)	1.00
Yes	19 (42.2)	11 (37.9)	0.84 (0.32, 2.17)
Alcohol dependence			
Low/moderate	3 (7.0)	1 (3.4)	1.00
High	40 (93.0)	28 (96.9)	0.47 (0.05, 4.82)
Illicit drug lifetime use			
No	259 (92.5)	230 (96.6)	1.00
Yes	21 (7.5)	8 (3.4)	2.33 (1.01, 5.36)
Illicit drug regular use			
No	8 (40.0)	2 (28.6)	1.00
Yes	12 (60.0)	5 (71.4)	0.60 (0.09, 3.88)
Illicit drug dependence			
Low	10 (50.0)	4 (57.1)	1.00
Moderate	2 (10.0)	2 (28.6)	0.40 (0.04, 3.90)
High	8 (40.0)	1 (14.3)	3.20 (0.29, 34.59)

After adjustment for the other individual characteristics, only under-graduation remained significantly associated with poor control of internet use with an adjusted odds ratio of 2.32 (Table 2). Furthermore, a dose-response relationship between under-graduation and an increased level of internet addiction was highlighted (Table 3).

**Table 2:** Adjusted odds ratios (OR) and 95% confidence intervals of reporting poor control of internet use by the individual characteristics among the participants from the colleges of Sousse, Tunisia in 2012-2013 school-year

**Characteristics**	**Internet addiction score**		**Adjusted OR (95% CI)** ^a^
	**≥50(n=280)**	**<50(n=238)**	
Age (yr)	21.5 (2.0)	22.1 (2.3)	0.98(0.86, 1.14)
Gender			
Female	139 (49.6)	136 (57.1)	1.00
Male	141 (50.4)	102 (42.9)	1.35(0.83, 2.19)
Field of study			
Health	55 (19.6)	62 (26.1)	1.00
Law	68 (24.3)	63 (26.5)	1.65 (0.84, 3.18)
Economy	33 (11.8)	22 (9.2)	1.07 (0.44, 2.54)
Technology	114 (40.7)	87 (36.6)	1.34 (0.71, 2.49)
Arts	10 (3.6)	4 (1.7)	2.45 (0.58,10.36)
Degree level			
Graduate degree	96 (34.5)	132 (55.5)	1.00
Undergraduate degree	182 (65.5)	106 (44.5)	2.32 (1.23, 4.35)
Academic success			
Yes	250 (89.6)	218 (91.6)	1.00
No	29 (10.4)	20 (8.4)	0.93 (0.46, 1.88)
Location			
Rural	220 (79.4)	184 (77.3)	1.00
Urban	57 (20.6)	54 (22.7)	1.27 (0.75, 2.16)
Single			
No	68 (24.5)	56 (23.6)	1.00
Yes	210 (75.5)	181 (76.4)	0.91 (0.57, 1.45)
Socioeconomic level			
High	25 (9.0)	19 (8.0)	1.00
Medium	240 (86.3)	212 (89.5)	0.87 (0.43, 1.76)
Low	13 (4.7)	6 (2.5)	1.76 (0.45, 6.82)
Mother is jobless			
No	78 (30.7)	59 (26.0)	1.00
Yes	176 (69.3)	168 (74.0)	0.87 (0.50, 1.51)
Father’s educational level		
Secondary school/ university level	43 (17.6)	57 (26.5)	1.00
Illiterate/ primary school	201 (82.4)	158 (73.5)	1.39 (0.80, 2.44)
Mother’s educational level		
Secondary school/ university level	100 (40.2)	117 (53.2)	1.00
Illiterate/ primary level	149 (59.8)	103 (46.8)	1.58 (0.94, 2.64)
Parental marital status		
Married	265 (96.7)	228 (97.9)	1.00
Divorced/widow	9 (3.3)	5 (2.1)	1.69 (0.43, 6.68)
Current residential status		
Alone or sharing with friends	71 (25.4)	62 (26.2)	1.00
With family	208 (74.6)	175 (73.8)	1.37 (0.83, 2.27)
Lifetime tobacco use			
No	212 (75.7)	198 (83.2)	1.00
Yes	68 (24.3)	40 (16.8)	1.44 (0.73, 2.84)
Alcohol use			
Lifetime use			
No	234 (83.6)	209 (87.8)	1.00
Yes	46 (16.4)	29 (12.2)	0.87 (0.39, 1.94)
Illicit drugs lifetime use		
No	259 (92.5)	230 (96.6)	1.00
Yes	21 (7.5)	8 (3.4)	1.17 (0.39, 3.50)

^a^Adjusted for all the variables in the table

**Table 3:** Internet addiction scores categories according to the individual characteristics among the participants from the colleges of Sousse, Tunisia in 2012-2013 school years

**Variables**	**No addiction** **n=42**	**Mild dependence** **n=196**	**Moderate dependence** **n=262**	**Severe dependence** **n=18**	***P*** **value**
Gender					0.190
Male	14 (33.3)	88 (44.9)	132 (50.4)	9 (50.0)	
Female	28 (66.7)	108 (55.1)	130 (49.6)	9 (50.0)	
Field of study					0.141
Health	14 (33.3)	48 (24.5)	54 (20.6)	1 (5.6)	
Law	15 (35.7)	48 (24.5)	63 (24.0)	5 (27.8)	
Economy	1 (2.4)	21 (10.7)	31 (11.8)	2 (11.1)	
Technology	10 (23.8)	77 (39.3)	105 (40.1)	9 (50.0)	
Arts	2 (4.8)	2 (1.0)	9 (3.4)	1 (5.6)	
Degree level					0.001
Graduate degree	30 (71.4)	102 (52.0)	93 (35.6)	3 (17.6)	
Undergraduate degree	12 (28.6)	94 (48.0)	168 (64.4)	14 (82.4)	
Academic success					0.128
Yes	42 (100.0)	176 (89.8)	236 (90.1)	14 (82.4)	
No	0 (0.0)	20 (10.2)	26 (9.9)	3 (17.6)	
Location					0.868
Rural	34 (81.0)	150 (76.5)	206 (79.5)	14 (77.8)	
Urban	8 (19.0)	46 (23.5)	53 (20.5)	4 (22.2)	
Single					0.823
No	10 (23.8)	46 (23.6)	62 (23.8)	6 (33.3)	
Yes	32 (76.2)	149 (76.4)	198 (76.2)	12 (66.7)	
Socioeconomic level					0.277
High	1 (2.4)	18 (9.2)	24 (9.2)	1 (5.6)	
Medium	41 (97.6)	171 (87.7)	225 (86.5)	15 (83.3)	
Low	0 (0.0)	6 (3.1)	11 (4.2)	2 (11.1)	
Mother is jobless					0.047
No	9 (22.5)	50 (26.7)	70 (29.3)	8 (53.3)	
Yes	31 (77.5)	137 (73.3)	169 (70.7)	7 (46.7)	
Father’s educational level					0.056
Secondary school/ university level	12 (33.3)	45 (25.1)	40 (17.5)	3 (18.8)	
Illiterate/ primary school	24 (66.7)	134 (74.9)	188 (82.5)	13 (81.3)	
Mother’s educational level					0.030
Secondary school/ university level	23 (60.5)	94 (51.6)	94 (40.2)	6 (40.0)	
Illiterate/ primary level	15 (39.5)	88 (48.4)	140 (59.8)	9 (60.0)	
Parental marital status					0.783
Married	41 (97.6)	187 (97.9)	249 (96.9)	16 (94.1)	
Divorced/widow	1 (2.4)	4 (2.1)	8 (3.1)	1 (5.9)	
Current residential status					0.285
Alone or sharing with friends	6 (14.3)	56 (28.7)	66 (25.3)	5 (27.8)	
With family	36 (85.7)	139 (71.3)	195 (74.7)	13 (72.2)	
Substances use					
Lifetime tobacco use					0.108
No	37 (88.1)	161 (82.1)	200 (76.3)	12 (66.7)	
Yes	5 (11.9)	35 (17.9)	62 (23.7)	6 (33.3)	
Tobacco use during the last month					0.330
No	1 (16.7)	4 (12.1)	8 (13.6)	0 (0.0)	
Yes	5 (83.3)	29 (87.9)	51 (86.4)	5 (100)	
Tobacco dependence					0.987
Low	4 (66.7)	22 (66.7)	35 (62.5)	4 (80.0)	
Moderate	2 (33.3)	10 (30.3)	19 (33.9)	1 (20.0)	
High	0 (0.0)	1 (3.0)	2 (3.6)	0 (0.0)	
**Alcohol use**					
Lifetime use					0.205
No	40 (95.2)	169 (86.2)	218 (83.2)	16 (88.9)	
Yes	2 (4.8)	27 (13.8)	44 (16.8)	2 (11.1)	
Regular use					0.354
No	1 (50.0)	17 (63.0)	25 (58.1)	1 (50.0)	
Yes	1 (50.0)	10 (37.0)	18 (41.9)	1 (50.0)	
Alcohol dependence					0.067
Low/moderate	0 (0.0)	3 (11.1)	4 (9.8)	0 (0.0)	
High	2 (100)	24 (88.9)	37 (90.2)	2 (100.0)	
Illicit drugs lifetime use					0.063
No	42 (100)	188 (95.9)	241 (92.0)	18 (100)	
Yes	0 (0.0)	8 (4.1)	21 (8.0)	0 (0.0)	
Illicit drugs regular use					0.001
No	0 (0.0)	5 (71.4)	12 (60.0)	0 (0.0)	
Yes	0 (0.0)	2 (28.6)	8 (40.0)	0 (0.0)	
Illicit drug dependence					0.354
Low	0 (0.0)	4 (57.1)	10 (50.0)	0 (0.0)	
Moderate	0 (0.0)	2 (28.6)	2 (10.0)	0 (0.0)	
High	0 (0.0)	1 (14.3)	8 (40.0)	0 (0.0)	

Even after binary logistic regression, the most influential risk factor on poor control of internet use was under graduation with an adjusted odds ratio of 2.45 (95% CI: 1.68, 3.57) (Table 4).

**Table 4:** Binary logistic regression analysis for socio-demographic characteristics related to poor control of internet use among participants from the colleges of Sousse, Tunisia in 2012-2013 school year (n=518)

**Characteristics**	**Unadjusted OR (95% CI)**	***P*** **value**	**Adjusted OR (95% CI)**	***P*** **value**
Degree level				
Graduate degree	1.00		1.00	
Undergraduate degree	2.36 (1.65, 3.36)	0.001	2.45 (1.68, 3.57)	0.001
Mother’s educational level			
Secondary school/ university level	1.00		1.00	
Illiterate/ primary level	1.69 (1.17, 2.44)	0.005	1.75 (1.20, 2.54)	0.004

## Discussion

The current study highlighted that 54% of participants showed features of internet addiction. Low parental educational levels, the younger age, lifetime tobacco use and lifetime illicit drug use were significantly associated with poor control of internet use among them. While the main predictor of poor control of internet use was under-graduation.

Compared to a recent multinational study led among medical students in 3 developing countries, the mean IAT score observed among participants was higher: 51.4 (±14.6) versus 31.4 (15.4). The student's repartition according to the 3 levels of internet addiction was also different: Among participants, 37.8% scored in the mild level category and 50.6% scored in the moderate level category of internet addiction versus 38.7 and only 10.5% in the multinational study. In the severe level of internet addiction category, 3.5% of participants were identified versus a smaller fraction of 0.5% in the multinational study ^21^. Otherwise, the prevalence of internet addiction among college students varies according to the countries, the cultural contexts, the tools and the cutoff point used to define the addiction levels. In Jordan for example, prevalence of poor control of internet use was 40% (using the IAT with a cutoff of 50) ^22^, in Egypt it was 13.2% (using the Young Internet Addiction Test with a cutoff point of 70) ^5^, in Turkey: 9.7% (using the 36 items Internet Addiction Scale) ^6^. In the United States and Europe, it ranged between 1.5% and 8.2% ^23^. In Tunisia, using Young's 8-item questionnaire a prevalence of 23.6% among Medical students ^24^ and a prevalence of 26.8% among Science students were reported ^25^. The high prevalence of internet use poor control among college students was explained by a need to escape the university stress and to develop new relationships ^5,26^. In Tunisia, the political instability triggered since 2011 because of the media blackout and the high level of unemployment among university graduates, might explain overuse of internet among young adults. Actually, internet represents a way to overcome geographic barriers and to broaden professional horizons.

The present study showed that younger students are more vulnerable to internet addiction^7,27^. In fact, due to their age-related features, they could be more interested than older students are in enjoyment and adventure that internet offers. The observation of a greater proportion of females scoring lower than 30 compared to males, corroborates several studies findings highlighting that males are at greater risk for developing internet addiction than females^8,21^. This was explained by a greater interest among males in sexual contents, technology, a lower parental supervision and more difficulties to make successful friendships than females^5,28^.

Under-graduation was the main predictor of poor control of internet use. Internet addiction is more common among freshman students due to their poor social relations as they are not familiarized yet with the new environment of the university^28,29^. Low parent's educational level was found to be another associated risk factor with poor control of internet use among especially among the mother. Parent's education level is directly related to the amount of internet use but inversely related to the rate of internet addiction ^28^. In fact, qualified parents may spend less time with their children ^30^ but on the other hand, they are more able to guide them to put the internet to good use^31^. Moreover, students whose parents had higher educational level and accordingly higher socioeconomic status might be less vulnerable to internet addiction due to their higher self-esteem ^32^.

Similarly, to another study showing association between internet addiction and substances use ^33^, poor control of internet use among participants was significantly associated with lifetime tobacco use and lifetime illicit drugs use. In addition, the association between internet addiction and illicit drugs use was stronger than its associations with tobacco use^33^, which joins the current study finding. Association between substances use and internet addiction could be explained by two facts: internet might serve as a channel to initiate substances use^34^. On the other side, the substance effect may increase the level of internet use and thus the risk of internet addiction^35^. While no significant association was found between alcohol use and internet addiction. The sedative effect of alcohol may explain this result^33^.

Unlike the previous studies led by Tunisia ^24,25^, the present study has the advantage of including college students from 5 different universities. Considering other concomitant addictive behaviors such as tobacco use, alcohol use and illicit drugs use represents another strong point of this study. However, the current results should be interpreted in the light of some limitations. Firstly, because of the cross-sectional nature of the study, it was not possible to report causal relationships but only simple associations. Another limitation was that data were self-reported which might result in under or over reporting addictive behaviors. While participants were asked out of classrooms and it was made clear to them that the questionnaire is anonymous and the participation is voluntary. Finally, for practical considerations, participants from each college were not randomly selected which could affect the sample representativeness. While, from each field of study, one college was randomly selected.

A national prevention program is required in Tunisia in order to reduce problematic internet use among youth. Early intervention targeting freshman students, self-esteem improvement and skills of life development may represent the key elements of internet addiction prevention among them. It is important also, to organize social activities at the campuses with the participation of the teachers as leaders in order to improve face-to-face interactions between students instead of virtual contact. Otherwise, monitoring software's and other technology based solutions could help in reducing the extreme forms of internet overuse. Reducing internet addiction might contribute to reducing substances use among them, as they are associated. Further national research among different age groups of youth is required to determine the most efficient time to intervene.

## Conclusions

Poor control of internet use is highly prevalent among the college students of Sousse especially those under graduate. A national intervention program would reduce this problem among youth. Further research is required to determine the national prevalence of internet addiction among both in school and out-of-school youth in Tunisia and to identify at-risk groups. This study will provide data used by health policymakers to address public health priority needs.

## Acknowledgements

The authors would like to thank the President of the University of Sousse and the Deans of the Institute of Fiscal Studies, the Institute of Music, the Faculty of Law, the Faculty of Medicine and the Higher Institute of Applied Sciences and Technology of Sousse.

## Conflict of interest statement

The authors of this paper have no conflict of interest.

## Funding

None.

## Highlights

- Poor control of internet use is highly prevalent among college students of Sousse, Tunisia
- Internet overuse among students in Sousse is associated with tobacco use
- Internet overuse among students in Sousse is associated with illicit substances use
- Internet overuse in Sousse students is inversely related to parent’s education level
- Undergraduate students are the most vulnerable group to internet addiction
